# Growing Degree-Day Measurement of Cyst Germination Rates in the Toxic Dinoflagellate Alexandrium catenella

**DOI:** 10.1128/aem.02518-21

**Published:** 2022-05-23

**Authors:** Alexis D. Fischer, Michael L. Brosnahan

**Affiliations:** a Biology Department, Woods Hole Oceanographic Institution, Woods Hole, Massachusetts, USA; Norwegian University of Life Sciences

**Keywords:** dinoflagellate cyst bed, population comparison, microbial resting stages, temperature-dependent phenology

## Abstract

Blooms of many dinoflagellates, including several harmful algal bloom (HAB) species, are seeded and revived through the germination of benthic resting cysts. Temperature is a key determinant of cysts’ germination rate, and temperature–germination rate relationships are therefore fundamental to understanding species’ germling cell production, cyst bed persistence, and resilience to climate warming. This study measured germination by cysts of the HAB dinoflagellate Alexandrium catenella using a growing degree-day (*DD*) approach that accounts for the time and intensity of warming above a critical temperature. Time courses of germination at different temperatures were fit to lognormal cumulative distribution functions for the estimation of the median days to germination. As temperature increased, germination times decreased hyperbolically. *DD* scaling collapsed variability in germination times between temperatures after cysts were oxygenated. A parallel experiment demonstrated stable temperature–rate relationships in cysts collected during different phases of seasonal temperature cycles *in situ* over three years. *DD* scaling of the results from prior A. catenella germination studies showed consistent differences between populations across a wide range of temperatures and suggests selective pressure for different germination rates. The *DD* model provides an elegant approach to quantify and compare the temperature dependency of germination among populations, between species, and in response to changing environmental conditions.

**IMPORTANCE** Germination by benthic life history stages is the first step of bloom initiation in many, diverse phytoplankton species. This study outlines a growing degree-day (*DD*) approach for comparing the temperature dependence of germination rates measured in different populations. Germination by cysts of Alexandrium catenella, a harmful algal bloom dinoflagellate that causes paralytic shellfish poisoning, is shown to require a defined amount of warming, measured in *DD* after cysts are aerated. Scaling by *DD*, the time integral of temperature difference from a critical threshold, enabled direct comparison of rates measured at different temperatures and in different studies.

## INTRODUCTION

During the termination phase of many dinoflagellate blooms, new resting cysts are formed and accumulate in bottom sediments (1). Blooms are revived through cyst germination, a process that produces single diploid planomeiocytes that undergo meiosis to yield new bloom-forming haploid vegetative cells (2). The timing and extent of germination are both critical for bloom initiation by many of these cyst-forming species (3–6).

Several conditions must co-occur for resting cysts to germinate: (i) cysts must be quiescent, a nondormant state in which they will germinate if environmental conditions are favorable (3, 7, 8); (ii) oxygen must be present (9, 10); and (iii) temperature must be within a suitable range (11, 12). In some species, light is also needed (13). When these prerequisites are met, temperature is the primary determinant of germination rate (14, 15). Temperature-germination rate relationships are therefore fundamental to understanding cyst bed persistence because they control a significant component of the total cyst loss rate. These relationships are also critically important to the timing and success of new bloom initiation.

**FIG 1:**
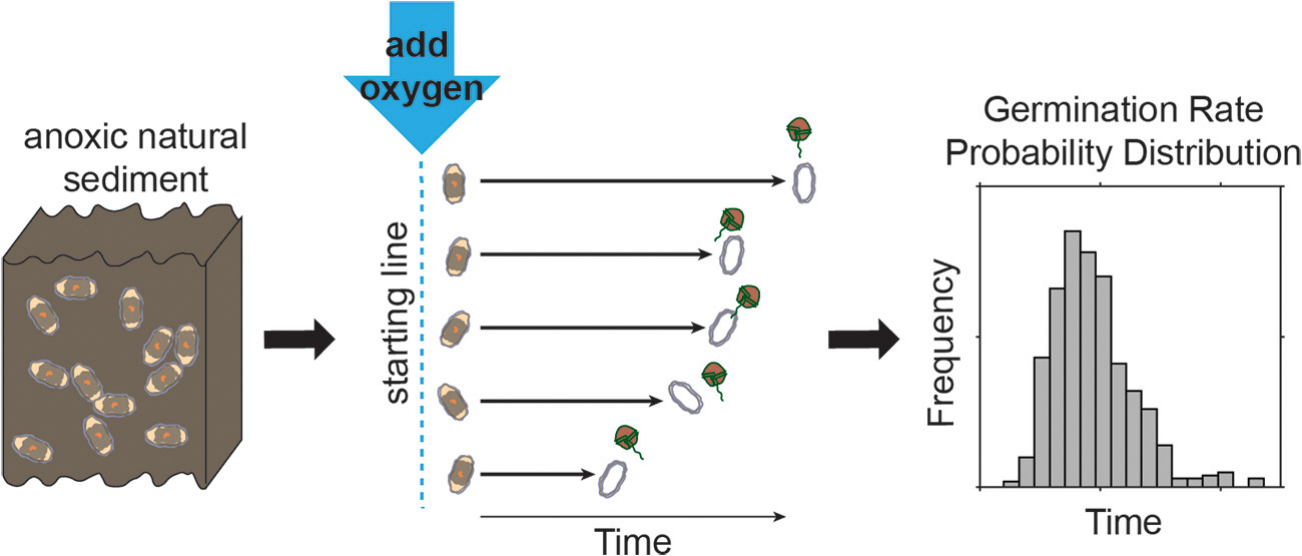
Schematic of how to measure germination rates of a natural population of A. catenella cysts. Aeration initiates the germination process, spurring quiescent cysts to germinate at their individual rates.

**FIG 2:**
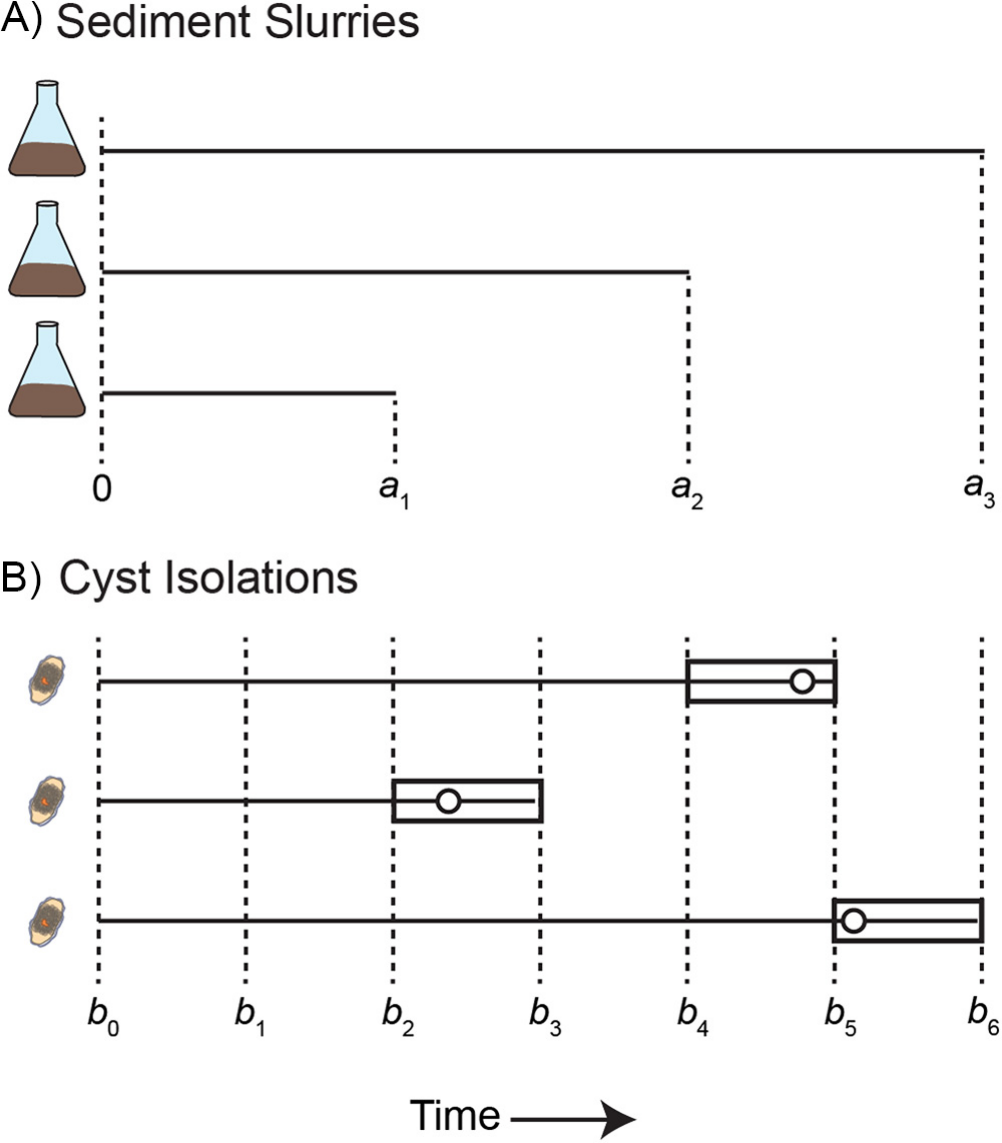
Observation schemes used in sediment slurry and cyst isolation-based germination rate measurements. (A) In sediment slurry experiments, replicate flasks are filled with approximately the same number of cysts at time zero. Flasks are incubated until removed for counting at a planned time *a*_i_. The difference between the number of cysts observed at time zero and *a*_i_ reflects the cumulative number of cysts that have germinated by time *a*_i_. (B) Isolation experiments track the germination of individual cysts through repeated observations by microscopy. Actual germination times are indicated by open circles, but observations only constrain germination time as occurring between times *b*_i–1_ and *b*_i_ (rectangles).

Cyst to cyst variability in the germination rate can be measured within natural populations by synchronizing germination through aeration of quiescent cysts collected from anoxic sediment (Fig. 1). The distribution of germination times (i.e., the period from a cyst’s activation by oxygen exposure to the production of a planomeiocyte) reflects the distribution of germination rates within a population. One common measurement method is replicate time course sampling of diluted cyst-rich “slurries” of natural sediment. Sediment samples containing large quantities of quiescent cysts are collected, then aerated through sediment processing and dilution. The resulting slurry is aliquoted to replicate vessels (e.g., flasks or tubes), which are incubated under controlled light and temperature conditions. Individual replicates are removed at defined time intervals and their remaining, ungerminated cysts are counted to reconstruct the germination time course (4, 10, 16) (Fig. 2A). Another method is repeated observation of single cysts. Single cysts are isolated into microwells of a tissue culture plate and each cyst is inspected for germination at regular intervals by microscopy (8, 14, 17) (Fig. 2B). Despite the common use of both slurry- and cyst isolation-based approaches, a comparison of results from dinoflagellate cyst experiments had not been reported when this study was begun.

Here, germination rates at different temperatures were measured using both the slurry and isolation approaches by cysts of Alexandrium catenella, a HAB species that causes paralytic shellfish poisoning (PSP), collected from the Nauset Marsh (Orleans, MA USA). The study applied growing degree-days (*DD*) to characterize and compare temperature–germination rate relationships. *DD*s are a common heuristic used to predict the phenology and development of terrestrial plants and insects, zooplankton, and even finfish (18–21), and have previously been used to accurately predict the phenology of inshore A. catenella blooms (22) and dormancy cycling of A. catenella cysts (5, 8). In this study, *DD* scaling shows strong overlap in median germination rates across several previously studied and widely dispersed cyst populations, demonstrating how the analytical approach described here can be used to compare rates measured through different studies and at different temperatures.

## RESULTS

### Slurry-based assessment of the temperature-germination rate relationship.

**FIG 3:**
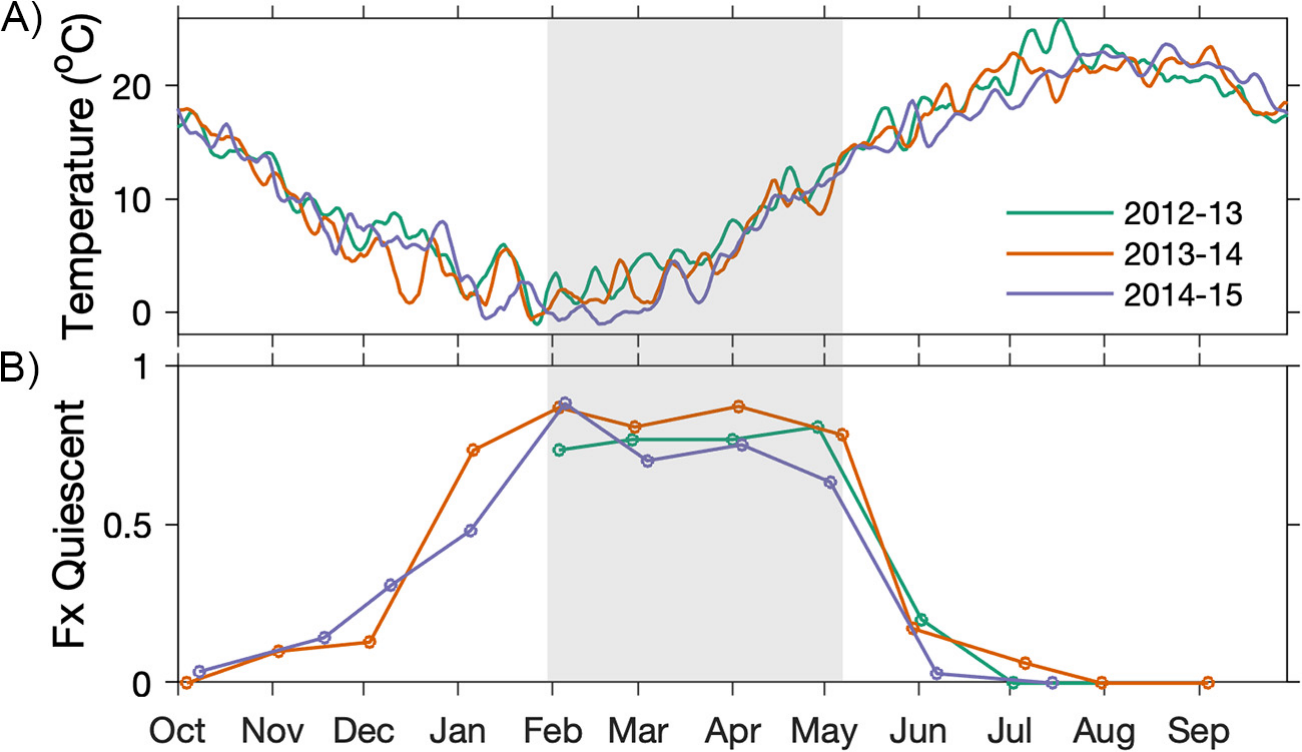
(A) Seasonal bottom water temperature in relation to (B) the quiescent fraction of the A. catenella cyst bed in Roberts Cove within Nauset from 2012 through 2015. A. catenella cysts cycle between states of quiescence (when they will germinate if exposed to oxygen and favorable temperatures) and dormancy (when they will not). In situ dormancy data are republished from cyst isolation experiments conducted by Fischer et al. (8). Each month, cohorts of freshly collected cysts were isolated and incubated under optimal conditions (oxygen, 15°C, light) and the quiescent fraction of the cyst bed was assessed from the cumulative fraction of cysts that germinated after 1 week. Gray shading demarcates the period when experiments in this study were conducted.

**FIG 4:**
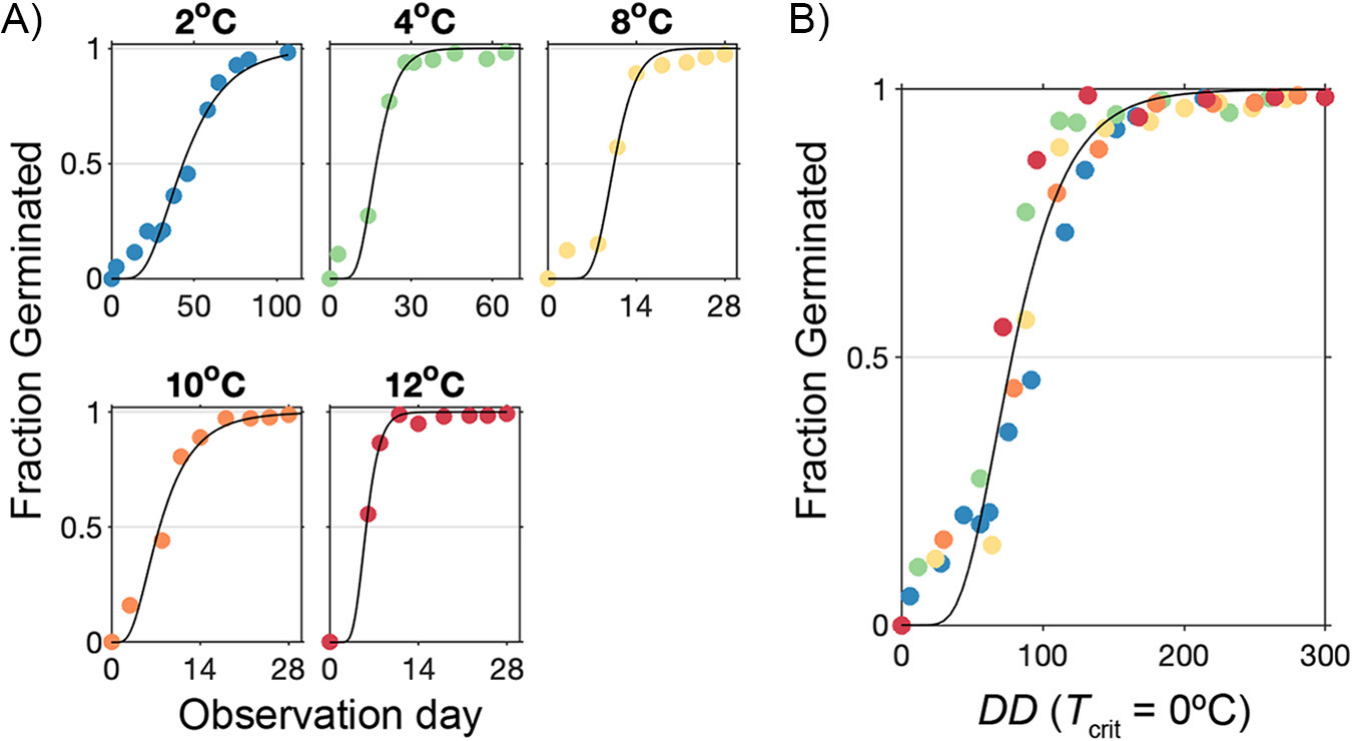
Cumulative germination of A. catenella cysts incubated in sediment slurries at different temperatures plotted as a function of (A) observation days and (B) *DD*. Data were fit to a lognormal CDF (black line) using maximum log-likelihood estimation.

Rate measurements were first evaluated through a large slurry experiment with *A. catenella* quiescent cysts collected in March 2014. The selected temperatures approximate the range of Nauset bottom water temperatures in the winter and spring when cysts are quiescent, and therefore germinable (8) (Fig. 3). Across all slurry experiments, germination rates increased with temperature through the full range tested (2, 4, 8, 10, and 12°C). This was most evident through comparisons of cumulative germination at early time points (Fig. 4A). For example, within the initial 8 days, cysts incubated at 8°C attained 15% cumulative germination, whereas cysts incubated at 12°C attained 87% cumulative germination. There was also an initial lag, or time delay before cysts began to germinate that scaled with temperature, i.e., there was a shorter lag at warmer temperatures and a longer lag at colder temperatures. For all temperature experiments, cysts had a mean maximum germination (*G*_max_) of 99% (*SD* = 0.6), demonstrating that essentially all cysts collected in March 2014 were viable and quiescent.

**FIG 5:**
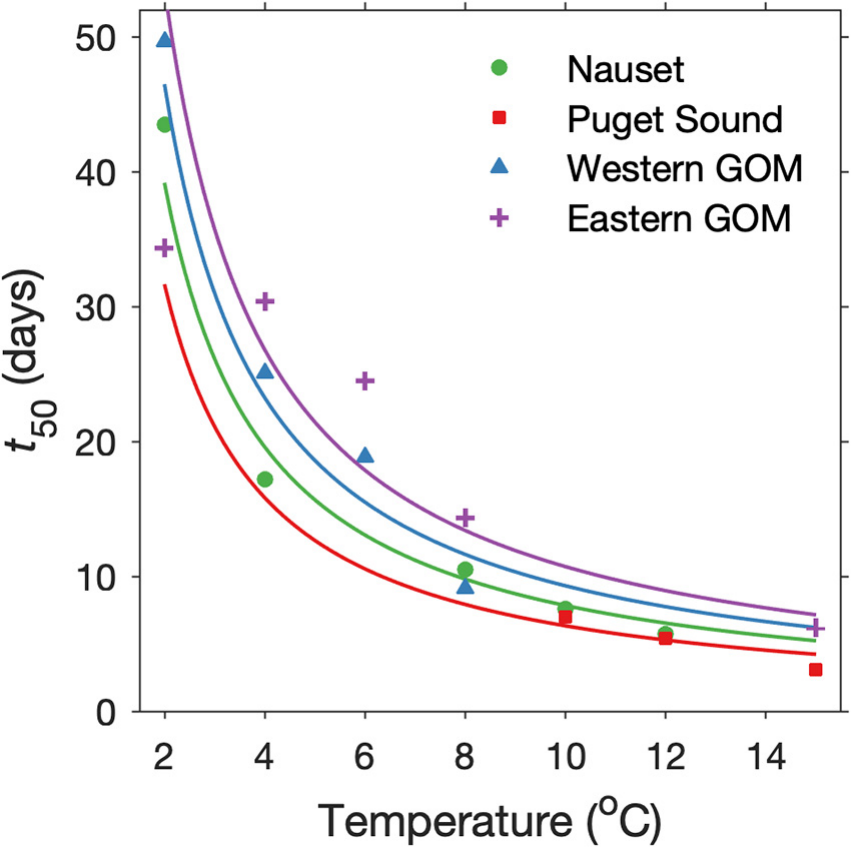
Days needed for A. catenella cysts to germinate at laboratory incubation temperatures. Data from Nauset cyst populations are shown alongside previously published data for cyst populations from Puget Sound and the western and eastern Gulf of Maine (GOM) (4, 16). All germination experiments were conducted with quiescent, subsurface (presumed anoxic) cyst populations that were aerated through the creation of slurries. The lines depict the degree-days to median germination (*DD*_50_) for each population. A similar plot was originally published in Anderson et al. (37) but not fully explained.

Fits of cumulative germination curves to lognormal cumulative distribution functions (CDFs) accurately predicted germination of all but the fastest cysts (Fig. 4). Curves were better defined in the lower temperature experiments than in higher ones due to a larger number of observations at intermediate levels of cumulative germination. For example, cumulative germination was less than 90% in nine time points in the 2°C experiments, but only in two time points in the 12°C experiments. Median germination times (*t*_50_) estimated from all fits were hyperbolically related to incubation temperature with values ranging from six days at 12°C to 44 days at 2°C (Fig. 5).

Scaling by *DD* was explored as a means to compare experiments performed at different incubation temperatures. Differences in procession towards complete germination collapsed after *DD* scaling (Fig. 4B) with remarkably low variance among *DD*_50_ estimates across individual temperature experiments (mean ± *SD*, 77 ± 8 DD). Similarly, lognormal fitting of all *DD*-scaled data produced a *DD*_50_ estimate of 78 DD. Analyses of residuals of the *DD*-scaled data to the respective lognormal distributions (individual and combined) also showed strong agreement with the lognormal CDF model, except at the lowest levels of cumulative germination where models tended to underestimate observed germination.

### Assessment of temperature–germination rate dependence on *in situ* conditioning.

**FIG 6:**
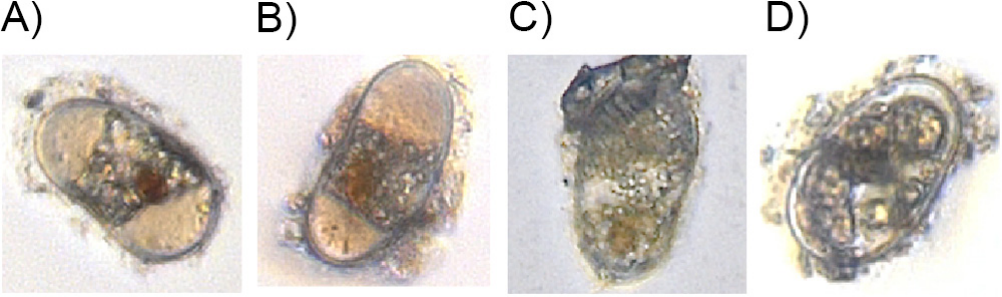
Example images of A. catenella cysts (A to C) with healthy appearances that were isolated at the beginning of isolation experiments and (D) a cyst that died during experimental incubation.

To assess whether recent environmental history might affect the distribution of germination times, isolation experiments measured the monthly germination rates of cysts collected from Nauset over three years during months when they were typically quiescent (February to May; 2013 to 2015; Fig. 3B). In these experiments, smaller samples of cysts were isolated from sediment samples, incubated at temperatures that were like those observed *in situ* during the same period, and their germination was monitored through weekly inspections (Fig. 2B). Much like slurry experiments, time to germination was inversely proportional to temperature but the maximum levels of cumulative germination were notably lower overall (mean *G*_max_ ± *SD*, 76% ± 9%). As a further comparison, the isolation experiment conducted with the same March 2014 sediment as the slurry experiments only reached a *G*_max_ of 62%. In contrast to the healthy appearance of cysts at the start of isolation experiments, cysts that did not germinate frequently turned black, displayed blebbing, and/or produced green autofluorescence at the end of experiments, all of which are morphological indicators of death (Fig. 6). Effects of the year and month of a cyst cohort on their *G*_max_ were tested through the application of a Pearson chi-squared test to data from 15°C control incubations. Neither differences between years (*X^2^* = 24, *n *= 12, *P* = 0.35) nor months (*X^2^* = 36, *n *= 12, *P* = 0.33) were significant, therefore, all monthly cyst collections were considered to have equivalent viabilities.

**FIG 7:**
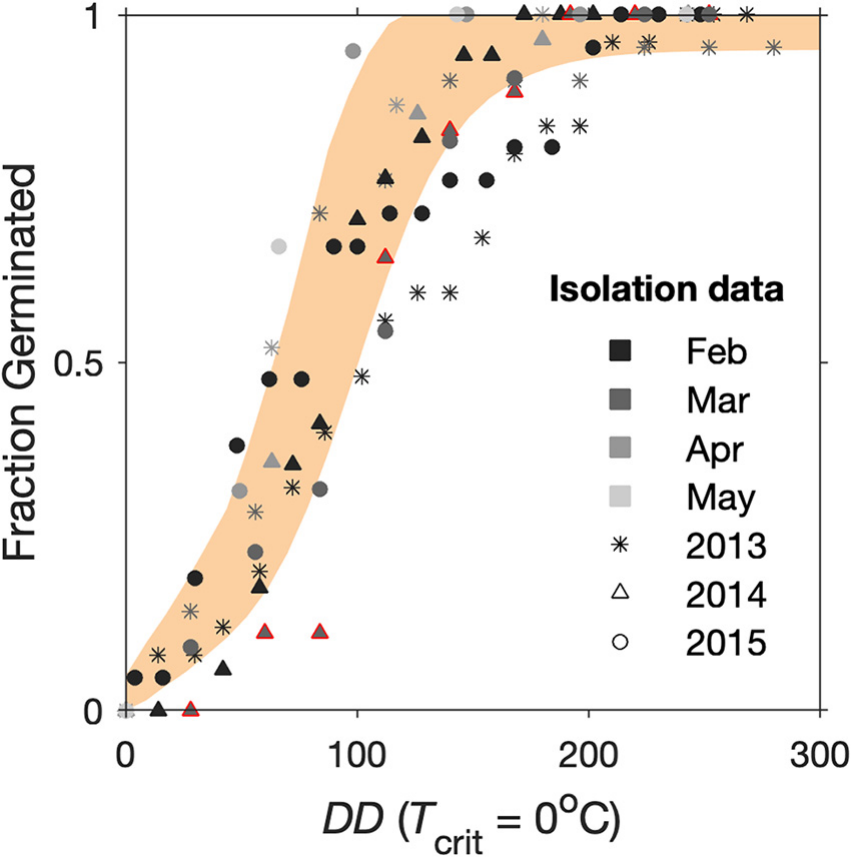
Cumulative germination of individually isolated A. catenella cysts incubated in different months and years at the *in situ* temperature. Different grayscale shades reflect the month and different shapes reflect the year that clutches of cysts were collected. For comparison, the range of cumulative germination observed in March 2014 by slurry experiments (Fig. 4B) is marked in orange, and the data points corresponding to the isolation experiment conducted at the same time are outlined in red.

No effects from different months or years of cyst collection were apparent after *DD* scaling of isolation experiment results. Estimates of cumulative germination generally overlapped those from the slurry-based temperature experiments (Fig. 7) and the *DD*_50_ from fitting to a single lognormal CDF distribution was 77 DD (*SD* = 19), essentially identical to estimates derived from slurry experiment data (Fig. 4). Given this consistency between slurry and isolation time courses, it was concluded that the distribution of germination times was insensitive to the seasonally changing environmental conditions experienced in Nauset.

As an initial assessment of whether *DD* scaling could be used to quantify temperature–germination rate relationship in other *A. catenella* populations, the same mathematical approach was applied to the previously published slurry experiments of Moore et al. (4) and Anderson et al. (16). The germination rate distributions of Puget Sound (WA, USA) and eastern and western Gulf of Maine (GOM) populations were found to be very similar to those from Nauset after *DD* scaling. In each case, germination time courses were well approximated using lognormal CDF functions and derived *t*_50_ values were hyperbolically related to incubation temperatures (Fig. 5). From the application of a common *T*_crit_ of 0°C, Puget Sound cysts appeared to require the fewest *DD* to germinate, followed by Nauset, western GOM, and eastern GOM cysts.

## DISCUSSION

This study successfully demonstrated a *DD*-based analytical approach for comparison of germination rates by A. catenella cysts measured at different temperatures and using different experimental approaches. Differences between respective temperature incubations were eliminated after *DD* scaling, revealing a highly consistent distribution of germination times among natural A. catenella cysts from Nauset throughout quiescent periods and over multiple years (Fig. 4 and 7). Likewise, *DD* scaling collapsed variability in cyst germination times from previously published studies of populations from Puget Sound and the GOM (Fig. 5). Consistent differences between the Nauset Marsh, Puget Sound, and GOM populations show how *DD* frameworks may facilitate the comparison of different populations, studies, and perhaps even different species. The results further show how *DD* relationships can improve the characterization of germination processes for a better understanding of the timing and magnitude of germination fluxes across different cyst habitats and in response to changing ocean bottom conditions.

### Stability of germination rates in a natural population.

The strong similarity of germination rates measured from quiescent A. catenella cysts sampled from Nauset in different months and years indicates that the rate distribution is a fixed characteristic of the population that does not respond to conditioning *in situ* (Fig. 4 and 7). Cysts measured through isolation experiments were different mixtures of year classes that experienced different conditions at the time of their formation and different temperature histories leading up to their collection for isolation experiments. Yet, no differences in the distribution of germination rates were apparent after *DD* scaling.

Also noteworthy was the strong similarity of estimates from the slurry- and isolation-based methods (Fig. 7). To our knowledge, this was the first study to compare slurry- and isolation-based germination rate measurements in a microbial eukaryote. The slurry design has several practical advantages relative to the isolation approach. These include the ability to make high frequency observations of germination more easily and to maintain consistent incubation temperatures throughout an experiment. In contrast, isolation-based measurements require repeated inspections of individual cysts, which can drive deviations from nominal incubation temperatures. Such deviations are an especially significant concern for the coldest incubations that take several months to complete.

This type of temperature stress may have contributed to observed differences in maximum cumulative germination observed between slurry and isolation experiments. Other notable differences between these approaches include sonication, density gradient enrichment, and micropipette manipulation of individual cysts. Prior applications of isolation-type methods to cysts from coastal GOM beds have yielded cumulative maximum germination levels exceeding 90% (7, 23), but these samples generally consist of much finer sediment than is present in Nauset (24). Higher fractions of coarse sand grains in Nauset sediment cores may have injured cysts during the initial sonication step of the isolation procedure, leading to reductions in their viability relative to slurry experiments (mean *G*_max_ ± *SD*, slurry = 99 ± 0.6%, isolation = 76 ± 9%). The significantly greater mortality in isolation experiments precluded a rigorous statistical comparison of the two methods, but they still produced nearly equivalent estimates of median germination time (77 and 78 DD for isolation and slurry approaches, respectively; Fig. 7). Future experiments applying repeated measures designs (like the isolation approach) should take care to document the timing and extent of cyst death through the full course of incubations or take steps to eliminate viability differences between methods (25). Limited divergence between the two methods was apparent only among the fastest germinating fraction of cysts. Specifically, a higher proportion germinated in the earliest stages of slurry experiments than was observed in the isolation experiments (Fig. 4 and 7).

Prior studies have observed sigmoid-type (S-shaped) time course patterns of cumulative germination in other A. catenella cyst populations (4, 10, 14, 16) and in many terrestrial plant seeds (26). Past A. catenella studies have attributed initial lags to an acclimation period associated with rapid changes in temperature or storage conditions (4, 16). It is clear from the present study that instead, lags reflect the relatively small proportion of fast germinating cysts in tested populations. *DD* scaling also enables consideration of the complete set of recorded germination times to extract estimates of mean or median germination rates. This contrasts with past studies that extracted linear segments of cumulative germination time courses (4, 16) or fit first-order exponential functions (10, 14) to estimate rates at different temperatures.

The *DD* framework also enables the description of the distribution of germination rates found in natural populations of cysts. Accounting for the full range of germination responses, from the fastest to the slowest cysts, can inform future studies that aim to quantify the *in situ* germling flux and the potential resilience of cyst populations faced with rapidly changing ocean bottom water conditions. Future work will be aimed at more rigorously fitting slurry data for pairwise statistical comparisons and for more accurate estimation of the full distribution of germination rates in different populations. The latter enables direct estimation of mean germination times expressed in *DD*, which is a more typical summary statistic for investigations of cyst bed behavior across a range of bottom water temperatures.

### Initial population comparisons.

Temperature–germination rate relationship comparisons across populations can have broad implications for species ecology and biogeography, and comparisons may also provide insights into how different populations will respond to a changing climate. Germination rate differences within and between populations can underlie a population’s ability to adapt and/or acclimate. However, at the time of this study, an approach to quantitatively compare cyst germination rates of different dinoflagellate populations did not exist, thus the framework provided by this study represents an important step forward.

The stability of germination rate distributions observed across years and different sampling months in Nauset suggests that the distribution is shaped by environmental selection on the population. In habitats that have large seasonal temperature shifts, larger *DD* requirements for germination could ensure that germlings emerge when conditions are more consistently favorable for bloom development. Thus, cysts would respond more slowly to short, unseasonable fluctuations in temperature, such as an anomalous warm spell during winter. Alternatively, in habitats with less seasonality that have shorter or irregular windows of favorable conditions for bloom development, selection may favor rapid germination so that cysts respond quickly to ephemeral opportunities for new bloom initiation.

*DD* scaling of germination time course data from other populations also demonstrates how this analytical approach may be used to compare results from different laboratories and choices of incubation temperature. The selected germination studies by Anderson et al. (16) and Moore et al. (4) were conducted over a decade apart, targeted different A. catenella populations, and investigated different incubation temperatures. Cumulative germination by all these populations and at all temperatures was well described by lognormal CDFs. Estimates of *t*_50_ were also hyperbolically related to incubation temperature (Fig. 5), such that median Puget Sound cysts appear to require the fewest *DD* to germinate, followed by those from Nauset and then GOM. Faster germination of Puget Sound cysts may reflect the relatively modest temperature seasonality of this system compared to Nauset and GOM. Habitats with weaker seasonality do not synchronize dormancy cycles to the same extent (5) and are associated with a long season of PSP risk (i.e., the potential for blooms) (27).

It remains to be shown if these observed germination differences are statistically significant, and if so, how differences in germination rates reflect differences in physical, chemical, or biological conditions across these and other globally distributed A. catenella habitats. Nauset and Puget Sound are both relatively shallow, inshore habitats where benthic cyst beds experience direct environmental signals associated with the onset of conditions supporting bloom development. In contrast, GOM cyst beds are in open coastal waters at depths that isolate them from conditions in overlying euphotic waters (14). Germling fluxes from each of these cyst beds are critical for the initiation of new blooms, and bloom timing and duration vary in ways that suggest a linkage to cyst bed activity (5, 6).

In many regions, warming bottom water temperatures are likely to drive dramatic changes in germling production from cyst beds (6). Therefore, several important questions that remain to be addressed include: (i) how may faster germination convey advantages in one habitat but not another?; (ii) which mechanisms drive selection for germination rates in different habitats, and over which time scales can populations shift in the face of rapidly changing coastal conditions?; and (iii) how and to what extent do populations retain diversity and variance among cysts with respect to their germination rates? To effectively connect populations’ germination physiology to biogeography, future work is needed to quantify population variance, to determine an approach to derive a population’s *T*_crit_ value for *DD* calculations, and to develop statistical approaches to compare derived parameters.

### Synchronization reveals degree-day relationships.

Synchronization through aeration of cysts was key to revealing the distribution of times required for individual cysts to germinate. Typical precautions were taken throughout sample collections for this study to prevent oxygen exposure before all cyst incubations. These included transport of cysts from the field in sediment cores and removal of any material that may have been exposed to air before the creation of sediment slurries or isolation of cysts to microwell plates. Because cysts only start the germination process after exposure to air, these precautions ensured that the initiation of germination was synchronized.

Inhibition of germination by anaerobiosis is by no means unique to A. catenella cysts. Diverse microbes and even aquatic animals produce similar resting stages and in many cases their germination or hatching requires oxygen. These include many species of dinoflagellates (9, 10), diatoms (28), cyanobacteria (29), and zooplankton (30, 31). For those species that are activated through oxygen exposure, a similar experimental approach may synchronize natural samples and enable the recording of germination or hatching response time distributions.

Also key to description of the cysts' germination rate distribution was the synchrony of their dormancy cycles. Many organisms experience dormancy, a physiological state that inhibits germination even when conditions are otherwise favorable. Control of dormancy cycles in *A. catenella* is via opposing temperature-dependent relationships that are well described by *DD* and a comparable chilling-unit function (5, 8). When the annual cycles of seasonal temperatures experienced by cysts are large, intervals of dormancy and quiescence tend to be phased. Inshore regions that experience especially cold wintertime temperatures are synchronized because cold exposure expedites passage through dormancy and then all but blocks passage through quiescence. This leaves cysts poised to respond to warming temperatures in spring (5). This seasonal synchronization of the Nauset cyst population also enables *DD*-based prediction of bloom timing (22).

Future experiments investigating the applicability of *DD* to physiological processes and phenology of other species should consider population synchronicity in their design and analysis. *DD*-type relationships will be obscured in processes that are not gated or otherwise synchronized in some way. Likewise, dormancy (in species where it occurs) may obscure or prevent measurements of germination time or other processes.

### Application for estimating *in situ* germling flux.

Germination is one of several temperature- and season-influenced processes that drive germling cell production from cyst beds. Other important processes include microbial respiration, sediment mixing, resuspension, and bioturbation, all of which control the exposure of buried cysts to oxygen and therefore the initiation of germination. Microbial respiration can be described by *DD* relationships in some systems (32) and increases in response to seasonal detrital inputs (33). Mixing processes, such as bioturbation and resuspension (by waves and currents), influences the vertical distribution of cysts within the sediment and the tortuosity of paths that germling cells must navigate to reach the water column (6, 24). Accurate estimation of fluxes must consider each of these processes and is therefore complex (34). Dinoflagellate germling fluxes are also challenging to measure *in situ*, so only a small number of observations have been reported to date (35, 36). This lack of data limits efforts to constrain the interacting effects of temperature on mixing, resuspension, and oxygen consumption on cyst germination and germling flux.

It is still possible to hypothesize cyst beds’ response to changing bottom water conditions under the assumption that fluxes are driven primarily by germination. Through the application of *DD* scaling, changes in the timing, intensity, and duration of cyst germination were recently estimated for a massive A. catenella cyst bed across the Chukchi Sea (37). The authors applied a generalized temperature–germination rate relationship from observations of several North American cyst populations. Due to observed changes in bottom water temperatures from 1999 to 2018, total germling production may have increased nearly two-fold and advanced up to three weeks earlier in the year. The latter phenological shift would represent a substantial expansion of the window for blooms to develop in this high latitude region. Still, other interacting effects of temperature could mitigate or magnify the direct effects of warming on germination rates. The potential scale of Chukchi blooms and the danger posed by PSP to human and animal populations calls for further investigations that link temperature change to cyst bed activity.

Uncertainty regarding how changing water temperatures may affect bioturbation, microbial respiration, and other benthic processes underscores the need for more studies that quantify their relationships with temperature. Each of these processes has a direct impact on the activity of many benthic resting stages that seed and shape the diversity and structure of plankton communities in overlying waters. The *DD* framework used in this study has already been useful in characterizing a wide range of biological phenomena. Many new applications are yet to be described in marine systems.

## MATERIALS AND METHODS

### Cyst collections from Nauset Marsh.

To isolate the effect of temperature on the germination rate, A. catenella cysts need to be both quiescent and anaerobically inhibited prior to the start of experiments. These conditions were met by collecting buried cysts and storing them in undisturbed sediment until experiments were begun. The proportion of cysts that were quiescent in each collection was assessed throughout the rate measurement experiments and through isolation-type assays as described by Fischer et al. (8).

All cysts were collected via sediment coring in Roberts Cove, a shallow area immediately adjacent to the southernmost of three drowned kettle holes within Nauset that host annual A. catenella blooms (38). Surficial cyst concentrations in this area are relatively high (typically >10^3^ cysts/cm^3^ in the 0 to 3 cm sediment layer) due to the heightened export of sexual stage cells during the termination of blooms (1). The bottom water temperature at the collection site was monitored for the duration of the study by a moored, internally recording HOBO logger (Onset Computer Corporation; Fig. 3A). Sediment cores were capped after collection and brought to our laboratory in Woods Hole, MA in an upright orientation, shielded from light, and maintained near their *in situ* temperature.

After sediment cores were brought to the laboratory, headwater was aspirated and cores were extruded. Cysts were collected from the sediment-water interface down to 3 cm. Because cyst concentrations within the 0 to 3 cm layer are generally greater subsurface (39, 40) and only a fraction of cysts from the top surface mm germinate from undisturbed sediment (40), most of the cysts collected for these experiments were presumed to be inhibited by anaerobiosis (10). The collected cysts were from several different year classes due to physical mixing and bioturbation processes in this environment.

### Germination rate measurements across different temperatures.

A large slurry experiment was used to measure the relationship between germination rate and temperature. In this design, the time course of germination is reconstructed from the loss of cysts observed in replicate cyst samples removed and counted at different time points (Fig. 2A). All cysts for the experiment were taken from a total of seven sediment cores, collected on 05 March 2014. These cores provided approximately 150 cm^−3^ of anoxic sediment, which was brought to a total volume of 1.2 L through the addition of an f/2 medium. The resulting slurry was homogenized and aliquoted in 10 mL volumes to 250-mL glass flasks (106 total), then diluted further through the addition of 50 mL of f/2 medium and swirled to mix and aerate as described by Anderson et al. (16). All manipulations were carried out inside a 4°C walk-in refrigerator to maintain the temperature observed in Nauset at the time of collection.

After aliquoting, a total of six flasks were immediately processed for counting to estimate the mean initial number of cysts in each of the aliquots. The remaining one hundred flasks were arrayed in sets of 20 in five different incubators set to 2, 4, 8, 10, and 12°C on a 14:10 light:dark cycle (150 μE/m^2^/s photon flux density). These temperatures approximate the range of Nauset bottom water temperatures in the winter and spring when cysts are quiescent, and therefore germinable (8) (Fig. 3). Sampling intervals varied between incubation temperatures to ensure adequate coverage of the germination time course, e.g., from 3 to 12 days at the coldest temperatures from 2 to 4 days at the warmest. At each sampling time point, flasks were removed from the experiment for counting. For most time points (~80%), flasks were removed and counted in duplicate, but occasionally only single flasks were taken to conserve samples. The remaining, uncounted slurry-filled flasks were thoroughly mixed once per week to provide comparable light exposure to all cysts.

The contents of slurry flasks were prepared for counting by primulin staining as described by Yamaguchi et al. (41). Cyst samples were fixed with formalin (5% vol/vol) at 4°C for at least 30 min and centrifuged (3000 × *g*, 5 min) before formalin was exchanged with cold methanol and stored at 20°C for at least 48 h for pigment extraction. Next, samples were centrifuged (3000 × *g*, 5 min) and methanol was exchanged with 10 mL distilled-deionized water. Samples were centrifuged again, and pellets were resuspended in a 2 mL staining solution (2.0 mg primulin/mL in distilled-deionized water). After staining for 1 h at 4°C on a laboratory shaker (Barnstead Thermolyne Labquake), samples were centrifuged, decanted, and the total volume was brought to either 5 or 10 mL with distilled-deionized water. The 5 mL resuspension volume was used for flasks processed at the end of the experiment that had fewer cysts. For each flask sample, all the cysts in a 1 mL subsample were counted under a Zeiss Imager microscope using blue light epifluorescence at 100× magnification with Zeiss filter set 09 (excitation 450 to 490 nm, dichroic 510 nm, emission 515 nm long pass). A. catenella cysts were identified by the “lime-green” fluorescence of their primulin-stain and their characteristic pill or capsule shape (55 to 60 μm long and 20 to 25 μm in diameter; Fig. 6). The fraction of cysts that germinated during each time interval was estimated as the difference between the mean initial cysts per flask and the mean cysts per flask remaining at each sampling time.

### Effect of temperature conditioning on the germination rate.

The question of whether differences in the recent environmental history (e.g., temperature) might affect the observed distribution of cysts’ germination rates was addressed through isolation-type experiments conducted over three years during months when Nauset cyst beds were nearly fully quiescent. Sediment for isolation experiments was collected in 2013, 2014, and 2015 during the first weeks of February, March, April, and May when cysts were known to be quiescent (Fig. 3B). A 5 cm^3^ subsample of anoxic surface sediment was disaggregated by sonication (Branson Sonifier 250; 1 min at 40%), and then passed through a series of Nitex sieves to isolate the cyst-containing 20 to 80 μm size fraction (42). The resulting sample was resuspended in 0.2 μm filtered seawater for further enrichment via density gradient centrifugation using a method described by Schwinghamer et al. (43). A. catenella cyst densities range from approximately 1.15 to 1.30 g/cm^3^ so a heavy cushion solution was prepared from colloidal silica (Nalco 1060, Nalco Chemical Co., Chicago, IL) and combined with sucrose to achieve a final density of 1.40 g/cm^3^. Cyst suspensions were underlaid with the colloidal silica suspension and then centrifuged at 1600 × *g* for 15 min at room temperature. Cysts were collected from the cushion/sediment suspension interface by pipette, then washed over a 20 μm Nitex sieve with 0.2 μm filtered seawater. Sieve contents were backwashed into a 15 mL centrifuge tube and 1 mL aliquots were pipetted into a Sedgewick-Rafter counting chamber from which cysts could be easily identified under a Zeiss Axioskop upright microscope at 100× magnification.

For each monthly germination assay, clutches of approximately 30 cysts with a healthy appearance (starch granules present, visible red eyespot, golden to brown coloration; Fig. 6A to C) were transferred by micropipette to individual wells of 96-well tissue culture plates and incubated at two temperatures: the approximate *in situ* bottom water temperature at the time of collection (2 to 12°C) and 15°C, an optimum control temperature at which most cysts will germinate within 1 week if viable and quiescent (Fig. 3) (8). From 2013 to 2014, the incubators used in April and May were discovered to be warmer than their nominal set points, ~9°C instead of 7°C and ~12°C instead of 11°C, but this was resolved by 2015. Our analysis was adjusted to reflect the true temperature experienced by cysts during their respective incubations.

Microwell plates were preloaded with 200 μL of modified f/2-Si medium and plates were sealed after cyst isolation to limit the evaporative loss of media (44). In all incubations, cysts experienced a 14:10 light:dark cycle (150 μE/m^2^/s^1^ photon flux density). Individual cysts were inspected for germination weekly under 100× magnification and scored as having germinated based on the presence of vegetative A. catenella cells or an empty cyst cell wall. As with the slurry experiment, the isolation experiment was terminated once no new germination had been observed for 2 consecutive weeks and a minimum of 4 weeks had elapsed. A small number of ungerminated cysts remained in the microwell plate at the termination of all isolation experiments. In comparison to the healthy appearances of cysts at the start of experiments, these remaining ungerminated cysts had unhealthy appearances (Fig. 6) and were, therefore, determined to have died. “Unhealthy” visual cues included blebbing, blackened coloration, and eyespots that were difficult to distinguish. Germinated cysts were divided by the total isolated cysts to calculate the maximum cumulative germination, G_max_, for each experiment. Results were compared to cumulative germination observed in the subset of slurry incubations in which no new germination was observed over at least the last 2 weeks of their incubation (temperatures >2°C).

### Fitting germination time course curves.

In analyzing germination time course data, it was important to consider sources of measurement uncertainty. For the slurry approach, data took the form of counts, and observational units (cyst samples/time points) were independent (Fig. 2A). In the isolation approach, the same cyst was repeatedly inspected at successive times, producing “time-to-event” type data that are commonly considered in survival analyses (Fig. 2B). In both approaches, the exact germination time of an individual cyst was not observed or recorded but instead occurs at some time before a final observation time point. Isolation experiments better constrain individual cyst germination times, but exact intervals were only known to be sometime between an experiment’s final and penultimate observations (i.e., interval-censored data).

Slurry and isolation-derived germination time course data were sigmoidal with time and were fit to several, common CDFs. Maximum likelihood estimation was used to fit Nauset time course data to several probability distribution models, including gamma, logistic, lognormal, and Weibull distributions. Of these, lognormal distribution best described cumulative germination data and was used to interpolate median germination times were required for subsequent analyses.

For the slurry data set, cumulative germination proportions at each time point were determined by dividing the remaining number of cysts by their initial number. For each experiment, cumulative germination was fit to a lognormal distribution using maximum likelihood estimation of the median with the nonlinear solver function *fminsearch* in MATLAB (MathWorks, Natick, MA).

Because cyst isolation data are interval-censored, a slightly different maximum likelihood model was applied that accounted for both minimum and maximum estimates of each cysts’ time to germination (25). Ideally, the status of each cyst at each time point would have been tracked with regard to four possible outcomes: viable-germinated, viable-ungerminated-quiescent, viable-ungerminated-dormant, or dead-ungerminated. However, cyst death was only recorded at the end of experiments, in part because the timing of cyst death is unclear. Visual cues of unhealthiness may develop some unknown time before or after a cyst expires (Fig. 6D). In other cases, no visual cue may be present that allows differentiation of viable, dormant cysts from those that have died. Some fraction of cysts collected from environmental samples inevitably expires due to their handling. We noted substantially higher cumulative germination in slurry experiments than in isolation experiments undertaken with material from the same set of sediment cores (05 March 2014) and consistently lower cumulative germination in isolation experiments overall. Based on these observations, we concluded that processing steps unique to the isolation experiment (e.g., sonication) likely injured a portion of the cysts. For this reason, cysts that had not germinated at the end of isolation trials were assumed to be dead and were discounted in estimates of cumulative germination. Censored data were then fit to a lognormal CDF distribution based on maximum likelihood with the *lognfitc* function in MATLAB (45).

### Degree-day scaling of the temperature–germination rate relationship.

As expected, warmer incubation temperatures drove faster germination. This was quantified by the median germination time or *t*_50_, which was estimated from lognormal fits of the respective temperature incubations. Because the relationship between temperature and *t*_50_ was hyperbolic, scaling by *DD* was explored to compare experiments performed at different incubation temperatures.

For each experimental time point, *DD* was calculated as the integral over time (*t*) of temperature above a threshold (*T*_crit_), $\mathrm{DD}\left(t\right)=\sum_{i=t_{0}}^{t}(T_{i}-T_{\mathrm{crit}})\Delta t$, where *t*_0_ is the starting time of the experiment, *T*_i_ is the daily temperature, and *T*_crit_ is the lower critical temperature below which germination does not occur. For each independent temperature experiment, the median degree-days to germination (*DD*_50_) was considered the most representative measure of a cyst cohort’s central tendency and was calculated as $DD_{50}=(T_{i}-T_{\mathrm{crit}})t_{50}$.

Because the lower physiological limit below which germination does not occur was unknown for the Nauset A. catenella cyst population, we explored T_crit_ values between −1 and 1°C. Values near 0°C minimized variance in *DD*_50_ across all temperatures tested in both slurry and isolation experiments. This temperature was therefore used for T_crit_ in all *DD* estimates from experiments conducted through this study.

### Preliminary application of *DD* scaling to other populations.

The same mathematical approach was applied to two previously published slurry experiments to assess if *DD* scaling was an effective means to quantify temperature–germination rate relationship in other *A. catenella* populations. The first study assessed germination rates at temperatures between 2 and 15°C by cysts from the open coastal regions of the western and eastern Gulf of Maine (GOM) (16), and the second at temperatures between 10 and 20°C by cysts from Bellingham Bay (Puget Sound, WA) (4). *DD* were calculated using *T*_crit_ of 0°C and results were fit to lognormal CDF distributions just as for the Nauset population to estimate *t*_50_ and *DD*_50_ values for the GOM and Puget Sound populations.
